# Patellofemoral Pain After Knee Trauma: Exploring Clinical, Biomechanical, and Muscle Strength Variables

**DOI:** 10.1111/sms.70274

**Published:** 2026-04-15

**Authors:** Helder dos Santos Lopes, Marina Cabral Waiteman, Fabio Viadanna Serrão, Joachim Van Cant, Bradley Neal, Ronaldo Valdir Briani, Fábio Mícolis de Azevedo

**Affiliations:** ^1^ School of Science and Technology, Sao Paulo State University (UNESP) Presidente Prudente Brazil; ^2^ Center of Biological Sciences and Health, Federal University of Sao Carlos (UFSCAR) Sao Carlos Brazil; ^3^ Faculté Des Sciences De La Motricité Humaine Université Libre De Bruxelles Brussels Belgium; ^4^ School of Sport, Rehabilitation and Exercise Sciences University of Essex Colchester UK; ^5^ Sports and Exercise Medicine Queen Mary University of London London UK

**Keywords:** kinematics, kinetics, knee injuries, musculoskeletal, patellofemoral pain syndrome

## Abstract

It remains unclear whether people with traumatic‐onset patellofemoral pain (PFP_T) present with the same physical and non‐physical changes as people with gradual‐onset patellofemoral pain (PFP_A). In this cross‐sectional study, we aimed to compare clinical, biomechanical and muscle strength outcomes between people with PFP_T, people with PFP_A, pain‐free controls with a history of knee trauma (CTRL_T), and pain‐free controls without a history of knee trauma (CTRL_A). Clinical variables included pain‐related characteristics, time since knee trauma, kinesiophobia, and functional capacity (e.g., self‐reported, objective). We captured kinematics and kinetics during a single‐leg landing task, and maximal isometric torque of the knee extensors and flexors, and the hip abductors using an isokinetic dynamometer. We performed between‐group comparisons using generalized linear models and independent *t*‐tests. The PFP_T group reported higher pain, greater kinesiophobia, and lower self‐reported function compared with the PFP_A group. The PFP_T group also demonstrated greater peak trunk flexion, greater peak hip flexion, and a lower knee contribution to the total support moment (TSM) compared with the PFP_A group. Both the PFP_T and PFP_A groups demonstrated reduced isometric peak torque of the knee extensors compared with the CTRL_T group. The CTRL_T group demonstrated higher peak hip flexion, lower knee contribution to TSM, and greater patellofemoral joint stress compared with CTRL_A. People with PFP_T demonstrated a worse clinical profile and a more hip‐dominant landing strategy compared with those with PFP_A. Most impairments (e.g., landing biomechanics and muscle strength) were evident only in comparison with CTRL_T, who also differed clinically and mechanically from CTRL_A.

## 1 Introduction

Patellofemoral pain (PFP) is characterized by diffuse anterior knee pain that worsens during weight‐bearing activities involving knee flexion, such as jumping and landing [1]. PFP is estimated to affect up to one‐quarter of the general population, particularly among young and physically active adults [2]. Symptoms often persist for decades, even after treatment [3, 4], and may be associated with an increased risk of developing knee osteoarthritis [5]. PFP also negatively impacts physical activity [1, 3], psychosocial well‐being [6], functional capacity [7], muscle strength [7, 8], and lower‐limb mechanics during dynamic tasks (e.g., hopping forward) [9, 10].

PFP is typically described as a gradual‐onset knee disorder [2, 11]. Yet, up to 70% of people who experience a traumatic knee event, such as blunt trauma and ligament tears, develop PFP symptoms that may persist for more than five years [12, 13]. People with traumatic‐onset PFP are commonly excluded from most studies [4, 7, 8, 11]. As a result, most of the available evidence on physical and non‐physical changes in people with PFP comes from studies focusing on those with gradual‐onset symptoms [6, 7, 9, 10]. It is currently unclear whether traumatic‐onset PFP presents with the same clinical profile (e.g., patient‐reported outcomes and objective function), trunk and lower limb biomechanics during dynamic tasks, and muscle strength deficits as gradual‐onset PFP. Similarly, little is known about how a history of knee trauma influences outcomes in pain‐free individuals, which may affect how research and clinical assessments are interpreted.

To address these gaps, we aimed to compare: (1) self‐reported pain, function, kinesiophobia, and performance during the single‐leg hop for distance (SLHD); (2) trunk and lower limb biomechanics during the SLHD; and (3) hip and knee muscle strength in people with PFP and a history of knee trauma (PFP_T), people with gradual‐onset PFP (PFP_A), pain‐free controls with a history of knee trauma (CTRL_T), and pain‐free without a history of knee trauma (CTRL_A). Given that both knee trauma and PFP may influence physical and non‐physical outcomes [14, 15], we hypothesized that the PFP_T group would present worse self‐reported pain, function, and greater kinesiophobia; a more hip‐dominant movement pattern (e.g., greater trunk and hip flexion); and reduced knee strength compared with the PFP_A group, as a result of cumulative effects.

## 2 Materials and Methods

We conducted a cross‐sectional study adhering to the Strengthening the Reporting of Observational Studies in Epidemiology (STROBE) guidelines [16], and reported according to the REPORTing of quantitative PatelloFemoral Pain (REPORT‐PFP) [17]. All procedures were approved by the university's Human Ethics Committee (approval number: 5.110.075), and all participants provided written informed consent before enrollment.

### 2.1 Participants

We recruited and assessed participants between November 2021 and December 2024 through advertisements at universities, fitness centers, local parks, and posts on social media (X and Instagram) in Presidente Prudente, Sao Paulo, Brazil. An experienced physiotherapist (> 7 years assessing people with PFP) evaluated participants for eligibility through a structured clinical assessment based on established diagnostic criteria for PFP [1], including symptom history, pain‐provoking activities, and exclusion of other knee pathologies. We used the following inclusion criteria for both groups with PFP (PFP_T and PFP_A): (i) symptoms exacerbated during at least two activities that load the patellofemoral joint (e.g., squatting, stair negotiation, running, jumping and/or landing); (ii) pain behind and/or around the patella for at least 3 months; and (iii) worst knee pain ≥ 20 mm on a 0–100 mm Visual Analog Scale in the previous month [1]. Additional criteria for PFP_T included a self‐reported and identifiable history of knee trauma (e.g., blunt trauma, patellar dislocation, ligament injuries and/or surgeries, and menisci injuries) that required medical attention and was perceived as the trigger for the onset of their PFP symptoms, with the absence of persistent symptoms other than PFP related to the traumatic event [12, 13, 14]. To minimize misclassification, we excluded participants from the PFP_T group if they reported any history of PFP symptoms before the traumatic event. We also excluded participants who were in the acute post‐traumatic phase (i.e., < 3 months). A history of knee trauma was an exclusion criterion for PFP_A. We included pain‐free controls if they had no signs or symptoms of PFP or other lower‐limb musculoskeletal disorders. CTRL_T participants had a history of knee trauma, whereas CTRL_A participants did not. When possible, medical reports and/or imaging exams were reviewed to corroborate the reported trauma. We did not include participants with other knee disorders (e.g., patellar tendon pathology, osteoarthritis), current low back pain or lower limb condition other than PFP, or cardiac/neurological conditions contraindicating participation.

We used G*Power (version 3.1, Heinrich‐Heine‐Universität Düsseldorf, Düsseldorf, Germany) to estimate the required sample size, with the stress in patellofemoral joint as the primary variable [9]. We specified an F test (ANOVA: fixed effects, omnibus, one‐way) with the following parameters: effect size of 0.32 (based on the stress results of a previous study) [18], four groups, an α of 0.05, and statistical power of 80%. This calculation indicated a total sample of 112 participants (28 per group).

### 2.2 Procedures

We collected data over two sessions, separated by at least two days and no more than seven days. We instructed the participants to refrain from sports or vigorous activities for 24 h before each session to minimize potential fatigue effects. All participants wore athletic shorts (and women additionally a sports top) to allow accurate marker placement. For participants with unilateral PFP, we tested the symptomatic limb; for those with bilateral symptoms, we tested the traumatic and/or most symptomatic limb. In pain‐free controls, we randomly assigned the tested limb by coin toss.

#### 2.2.1 First Day

We initially collected anthropometrics data (body mass, height, body mass index [BMI]), and self‐reported measures. We assessed pain severity using a 0–100 mm visual analogue scale. We asked participants to report the worst pain experienced in the previous month. We also asked participants to report the duration of PFP symptoms and time since knee trauma, both recorded in months. Kinesiophobia was assessed using the Tampa Scale for Kinesiophobia [19], and self‐reported function was assessed using the Knee Injury and Osteoarthritis—Patellofemoral subscale [20], with the total score being used in the analyses. For biomechanical evaluation, we measured knee and ankle widths using a universal caliper (150 mm with 0.02 mm precision; Digimess), lower‐limb length (anterior superior iliac spine to lower border of the medial malleolus) using a tape measure (0–150 mm), and placed 23 reflective markers (14 mm) at specific anatomical points according to the Plug‐in‐Gait marker set: 7th cervical vertebra, 10th thoracic vertebra, jugular notch, xiphoid process, and over the right scapula. For the pelvis and lower limbs, we positioned markers on the right and left anterior superior iliac spines, posterior superior iliac spines, lateral thighs, medial (additional) and lateral knees, lateral malleoli, second metatarsal heads, and the posterior aspect of the heel.

We performed motion capture using an eight‐camera system (five Bonita 10 and three Vero cameras from VICON Movement Systems Inc., USA) sampling at 100 Hz. We collected a static trial with the participants standing on a force plate (Bertec Corporation, Columbus, OH, FP4060) synchronized with the motion capture system. We collected kinetic data at a sampling frequency of 1000 Hz. We used a virtual knee alignment device (KAD) during the static calibration to improve determination of the knee joint center.

We collected kinematic and kinetic data during a single‐leg hop for distance. We instructed participants to perform the task at maximal effort, with their hands on their waist, and to land on the force plate. Before data collection, participants performed three familiarization trials. For analysis, we used the average of three successful trials with full‐foot contact [21]. We normalized the hop distance to limb length ([cm/cm] × 100) [22].

#### 2.2.2 Second Day

We assessed isometric torque of the knee extensors, flexors, and hip abductors using an isokinetic dynamometer (Biodex Multi‐Joint System 4 Pro, Biodex Medical Incorporation, New York, NY, USA) sampling at 100 Hz. The same evaluator performed all assessments, with the order of muscle groups randomized using sealed opaque envelopes. Before testing, participants performed a standardized dynamic warm‐up consisting of agonist and antagonist movements (20 submaximal repetitions at 180°/s) for each joint (i.e., knee extension/flexion and hip abduction/adduction). Participants also performed two submaximal familiarization contractions, at up to 60% of their perceived maximal effort (6 s each) [8]. For testing, participants performed three maximal voluntary isometric contractions (6 s) with 1‐min rest period intervals, receiving standardized verbal encouragement and visual feedback. We calculated the average of the three trials to represent the maximal isometric torque and normalized it to body mass (Nm·kg^−1^ × 100 = % body mass) [8].

For knee extensors and flexors, we positioned participants seated with the hip at 90° and knee at 60°, and the trunk and thigh stabilized with straps [8]. We aligned the dynamometer axis of rotation to the lateral epicondyle of the femur, and the lever arm five cm above the lateral malleolus [23]. For hip abductors, we positioned the participants in a side lying position with the tested limb on top, hips in neutral, and the trunk and contralateral limb stabilized with straps. We aligned the dynamometer axis of rotation to the intersection of one line from the posterior‐superior iliac spine toward the knee and another line from the greater trochanter of the femur toward the midline of the body [23]. We positioned the lever arm five cm above the base of the patella [8]. We instructed participants not to bend their knees during the test and assessed the isometric torque at 20° of hip abduction [23].

### 2.3 Data Analysis

We processed marker trajectories in ViconNexus 2.9 (Vicon Motion Systems Ltd). We filtered trajectories with a Woltring filter (root mean square error of 2 mm) to reduce noise due to soft tissue artifact. We determined knee and ankle joint centers as previously described [24], and hip joint center using the Bell, Pedersen, and Brand method [25]. We calculated the Euler angles based on the joint coordinate system definitions recommended by the International Society of Biomechanics for static calibration [26]. We expressed the kinematics of the hip, knee, and ankle as distal relative to proximal segments, and trunk kinematics relative to the laboratory coordinate system.

We computed net joint moments using inverse dynamics equations and normalized to body mass. We calculated the biomechanical variables of interest with custom MATLAB scripts (Version R2023a; The MathWorks Inc., Natick, MA). Only the landing phase of the SLHD was considered for analysis. The landing phase was defined from initial contact (vertical ground reaction force exceeded 10 N) to peak knee flexion [27]. We extracted peak flexion angles (trunk, hip, knee, and ankle), as well as peak hip and knee extensor and ankle plantarflexor moments. We used joint moments to calculate the total support moment (TSM) and each joint's relative contribution [28].

We calculated patellofemoral joint (PFJ) stress using the biomechanical model of the knee proposed by Devita & Hortobagyi (2001) [29]. This model uses data from hip, knee, and ankle angles and their respective joint moments to obtain the muscle forces of hamstrings, quadriceps femoris, and triceps surae. This allows the calculation of the knee extensor moment adjusted by the co‐contraction of the knee flexors, which could underestimate PFJ stress if not considered. We calculated the PFJ stress throughout the landing phase of the SLHD, but the peak was our parameter of interest. The complete description of the model can be found elsewhere [29].

### 2.4 Statistical Analysis

We summarized participant and group characteristics based on demographic and self‐reported outcomes. We compared demographics, self‐reported, trunk and lower limb biomechanics, and muscle strength outcomes between groups using Generalized Linear Models or independent *t*‐tests, where appropriate. For Generalized Linear Models, we selected linear, gamma, or tweedie distributions based on goodness‐of‐fit indices (e.g., AIC, BIC, or deviance). We included sex as a covariate in the analysis of SLHD distance, trunk and lower limb biomechanics, and muscle strength. For post hoc pairwise comparisons, we applied the least significant difference method. Before independent *t*‐tests, we assessed data normality and homogeneity of variance using the Shapiro–Wilk test and Levene's test, respectively. When the assumption of equal variances was violated, we applied Welch's *t*‐test. We calculated effect sizes (Cohen's *d*) and the confidence intervals (CI) and interpreted them as no effect (< 0.19), small (0.20–0.49), medium (0.50–0.79), and large (≥ 0.80) [30]. We performed all statistical analyses using the Statistical Software for Social Sciences (IBM SPSS Statistics for Windows; v. 29.0; IBM Corp), and set the statistical significance at *p* < 0.05. Detailed pairwise comparisons between groups are provided in the Table S1.

## 3 Results

### 3.1 Participant Characteristics

**FIGURE 1:**
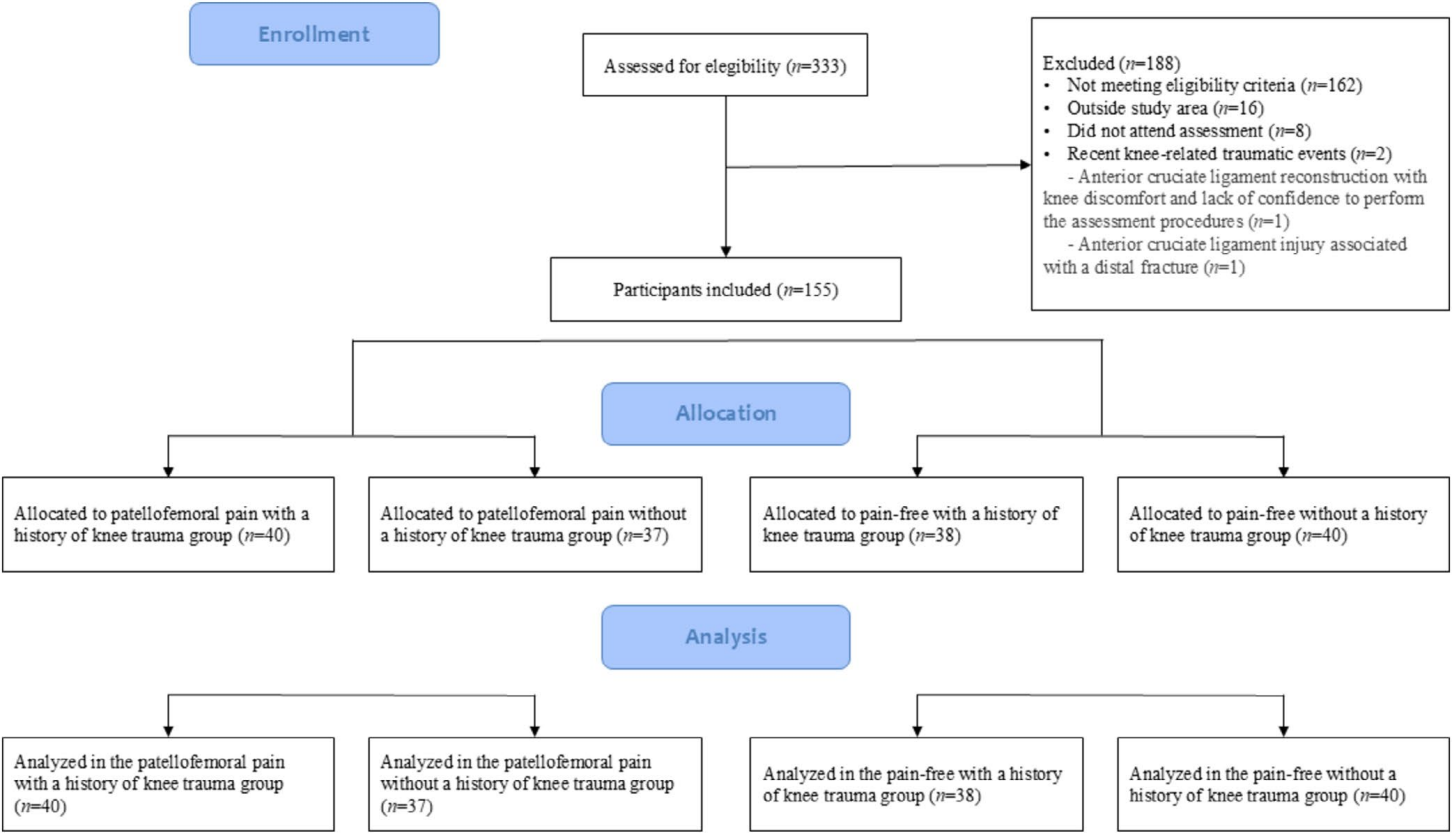
Participant flow diagram.

Of the 333 people who initially expressed interest in the study (Figure 1), we excluded 188 due to failure to meet the eligibility criteria (*n* = 162), residence outside the study region (*n* = 16), failure to attend the scheduled assessments despite previous appointment (*n* = 8), and the presence of recent knee‐related traumatic events (< 1 month; *n* = 2). We included a total of 155 participants (response rate = 83.2%). We grouped participants as follows: PFP_T (*n* = 40), PFP_A (*n* = 37), CTRL_T (*n* = 38), and CTRL_A (*n* = 40).

We identified no significant differences between PFP_T and PFP_A for age, body mass, height, or BMI (*p* > 0.05; Table 1). Compared with controls, PFP_T participants presented with a higher BMI compared with CTRL_T (*p* = 0.015; 95% CI [0.47, 4.17]; *d* = 0.57) and CTRL_A (*p* = 0.031; 95% CI [0.21, 4.19]; *d* = 0.49). In the PFP_A group, participants were shorter than CTRL_T (*p* = 0.004; 95% CI [−9.13, −1.77]; *d* = −0.68), but did not differ significantly from CTRL_A (*p* > 0.05). CTRL_T participants were taller than CTRL_A (*p* = 0.018; 95% CI [0.78, 8.10]; *d* = 0.55). Characteristics of knee trauma in the PFP_T and CTRL_T groups, including type and timing of knee injury or surgery, are summarized in Tables 1 and 2.

**TABLE 1:** Participants' characteristics.

Variables	Groups			
	PFP_T (*n* = 40)	PFP_A (*n* = 37)	CTRL_T (*n* = 38)	CTRL_A (*n* = 40)
*Demographics*				
Sex, females/males	20/20	23/14	9/29	18/22
Age, years	23.75 ± 4.37	23.16 ± 3.56	23.29 ± 4.89	22.50 ± 3.80
Body mass, kg	76.45 ± 16.98	69.04 ± 16.24	72.65 ± 10.62	70.11 ± 15.97
Height, cm	170.12 ± 9.00	168.35 ± 8.04^c^	173.80 ± 7.94^b^, ^d^	169.36 ± 8.29^c^
Body mass index, kg/m [2]	26.37 ± 4.89^c^, ^d^	24.30 ± 5.20	24.05 ± 3.06^a^	24.17 ± 4.03^a^
*Type of knee injury/surgery*				
ACL partial injury, %	2 (5)	NA	4 (10.53)	NA
ACL reconstruction with hamstring graft, %	3 (7.5)	NA	6 (15.79)	NA
ACL reconstruction with patellar tendon graft, %	1 (2.5)	NA	2 (5.26)	NA
Blunt trauma, %	11 (27.5)	NA	15 (39.47)	NA
Knee sprain, %	9 (22.5)	NA	3 (7.89)	NA
Lateral meniscus injury, %	4 (10)	NA	2 (5.26)	NA
LCL partial injury, %	0 (0)	NA	1 (2.63)	NA
Menisci injury, %	1 (2.5)	NA	0 (0)	NA
Multiple ligaments injury (ACL and MCL), %	1 (2.5)	NA	0 (0)	NA
Patellar dislocation, %	8 (20)	NA	5 (13.16)	NA

*Note:* Data are reported as mean ± SD or *n* (%) unless otherwise indicated.

Abbreviations: ACL, anterior cruciate ligament; LCL, lateral collateral ligament; MCL, medial collateral ligament; NA, not applicable. Both PFP_T and CTRL_T groups included a heterogeneous range of knee injuries and surgical procedures. Trauma categories are presented descriptively and were not used for subgroup analyses.

^a^ Represents significant difference as compared with PFP_T.

^b^ Represents significant difference as compared with PFP_A.

^c^ Represents significant difference as compared with CTRL_T.

^d^ Represents significant difference as compared with CTRL_A.

**TABLE 2:** Clinical variables.

Variables	Groups			
	PFP_T (*n* = 40)	PFP_A (*n* = 37)	CTRL_T (*n* = 38)	CTRL_A (*n* = 40)
Worst pain in the last month, VAS	54.25 ± 18.06^b^	44.32 ± 18.45^a^	NA	NA
Duration of PFP symptoms, months	52.47 ± 37.61	50.68 ± 52.72	NA	NA
Time since knee trauma, months	57.45 ± 34.89	NA	61.18 ± 48.96	NA
Kinesiophobia, TSK	34.89 ± 5.65^b^, ^c^, ^d^	30.81 ± 6.63^a^	30.24 ± 5.83^a^	29.55 ± 6.47^a^
Self‐reported function, KOOS‐PF	64.30 ± 17.01^b^, ^c^, ^d^	73.92 ± 15.45^a^, ^c^, ^d^	93.65 ± 8.75^a^, ^b^, ^d^	97.97 ± 4.17^a^, ^b^, ^c^
Single‐leg hop distance, (cm/cm) × 100	101.72 ± 22.03^c^, ^d^	112.11 ± 35.48^c^	128.10 ± 27.27^a^, ^b^, ^d^	115.53 ± 25.77^a^, ^c^

*Note:* Data are reported as mean ± SD.

Abbreviations: VAS, Visual Analog Scale; TSK, Tampa Scale for Kinesiophobia; KOOS‐PF, Knee injury and Osteoarthritis Outcome Score—Patellofemoral subscale; cm, centimeters.

^a^ Represents significant difference as compared with PFP_T.

^b^ Represents significant difference as compared with PFP_A.

^c^ Represents significant difference as compared with CTRL_T.

^d^ Represents significant difference as compared with CTRL_A.

### 3.2 Clinical Outcomes

PFP_T participants reported higher pain in the last month (*p* = 0.020; 95% CI [1.64, 18.22]; *d* = 0.54), greater kinesiophobia (*p* = 0.005; 95% CI [1.29, 6.87]; *d* = 0.66), and lower self‐reported function (*p* = 0.012; 95% CI [−17.02, −2.22]; *d* = −0.59) compared with PFP_A participants, with no significant differences in symptom duration and SLHD performance (*p* > 0.05; Table 2). Compared with CTRL_T participants, PFP_T participants presented with greater kinesiophobia (*p* = 0.001; 95% CI [2.06, 7.24]; *d* = 0.81), lower self‐reported function (*p* < 0.001; 95% CI [−35.50, −23.20]; *d* = −2.15), and lower SLHD performance (*p* < 0.001; 95% CI [−37.53, −15.23]; *d* = −1.07), with no significant differences for time since knee trauma (Welch's *t*‐test, *p* > 0.05). Compared with CTRL_A participants, PFP_T participants presented with greater kinesiophobia (*p* < 0.001; 95% CI [2.64, 8.04]; *d* = 0.88), lower self‐reported function (*p* < 0.001; 95% CI [−39.18, −28.16]; *d* = −2.72), and lower SLHD performance (*p* = 0.012; 95% CI [−24.48, −3.14]; *d* = −0.58). Compared with CTRL_T participants, PFP_A participants presented with lower self‐reported function (*p* < 0.001; 95% CI [−25.49, −13.97]; *d* = −1.58) and lower SLHD performance (*p* = 0.032; 95% CI [−30.53, −1.45]; *d* = −0.51), with no significant differences for kinesiophobia (*p* > 0.05). Compared with CTRL_A participants, PFP_A participants presented with lower self‐reported function (*p* < 0.001; 95% CI [−29.10, −19.00]; *d* = −2.16). No significant differences were identified for SLHD performance and kinesiophobia (*p* > 0.05). Compared with CTRL_A participants, CTRL_T participants presented with a lower self‐reported function (*p* = 0.006; 95% CI [−7.39, −1.25]; *d* = −0.64) and higher SLHD performance (*p* = 0.040; 95% CI [0.61, 24.53]; *d* = 0.47). No significant differences were identified for kinesiophobia (*p* > 0.05).

### 3.3 Kinematics

PFP_T participants demonstrated greater trunk flexion (*p* = 0.034; 95% CI [0.41, 10.07]; *d* = 0.49), and hip flexion (*p* = 0.030; 95% CI [0.57, 10.91]; *d* = 0.50) compared with PFP_A participants, with no significant differences in knee flexion and ankle dorsiflexion (*p* > 0.05; Table 3). Compared with controls, PFP_T participants demonstrated no significant differences in trunk flexion, hip flexion, knee flexion, and ankle dorsiflexion (*p* > 0.05). Compared with CTRL_T participants, PFP_A participants demonstrated lower trunk flexion (*p* = 0.013; 95% CI [−10.28, −1.24]; *d* = −0.59), and hip flexion (*p* = 0.030; 95% CI [−12.08, −0.64]; *d* = −0.51), but no significant differences when compared with CTRL_A participants (*p* > 0.05). No significant differences were identified for knee flexion and ankle dorsiflexion (*p* > 0.05). Compared with CTRL_A participants, CTRL_T participants demonstrated greater hip flexion (*p* = 0.048; 95% CI [0.04, 10.00]; *d* = 0.46). No significant differences were identified for trunk flexion, knee flexion, or ankle dorsiflexion (*p* > 0.05).

**TABLE 3:** Trunk and lower limb biomechanics outcomes.

Variables	Groups			
	PFP_T (*n* = 40)	PFP_A (*n* = 37)	CTRL_T (*n* = 38)	CTRL_A (*n* = 40)
Peak trunk flexion, deg	21.53 ± 10.56^b^	16.29 ± 10.71^a^, ^c^	22.05 ± 8.88^b^	18.47 ± 11.13
Peak hip flexion, deg	52.04 ± 9.93^b^, ^d^	46.3 ± 12.77^a^, ^c^	52.66 ± 12.08^b^	47.64 ± 9.93^a^
Peak knee flexion, deg	46.87 ± 10.75	45.38 ± 11.86	50.86 ± 12.51	48.75 ± 10.75
Peak ankle dorsiflexion, deg	19.25 ± 6.32	18.71 ± 6.63	19.41 ± 7.09	20.49 ± 6.32
Total support moment, Nm·kg^−1^	7.87 ± 1.96^c^	7.81 ± 3.04	8.88 ± 1.97^a^, ^d^	7.96 ± 1.64^c^
Peak patellofemoral joint stress, MPa	22.63 ± 6.96^c^	21.30 ± 11.68^c^	26.72 ± 10.42^a^, ^b^, ^d^	21.69 ± 7.27^c^

*Note:* Data are reported as mean ± SD. Data were analyzed using generalized linear models adjusted for sex.

Abbreviations: deg., degrees; MPa, megapascal; Nm·kg^−1^, Newton‐meters per kilogram.

^a^ Represents significant difference as compared with PFP_T.

^b^ Represents significant difference as compared with PFP_A.

^c^ Represents significant difference as compared with CTRL_T.

^d^ Represents significant difference as compared with CTRL_A.

### 3.4 Kinetics

**FIGURE 2:**
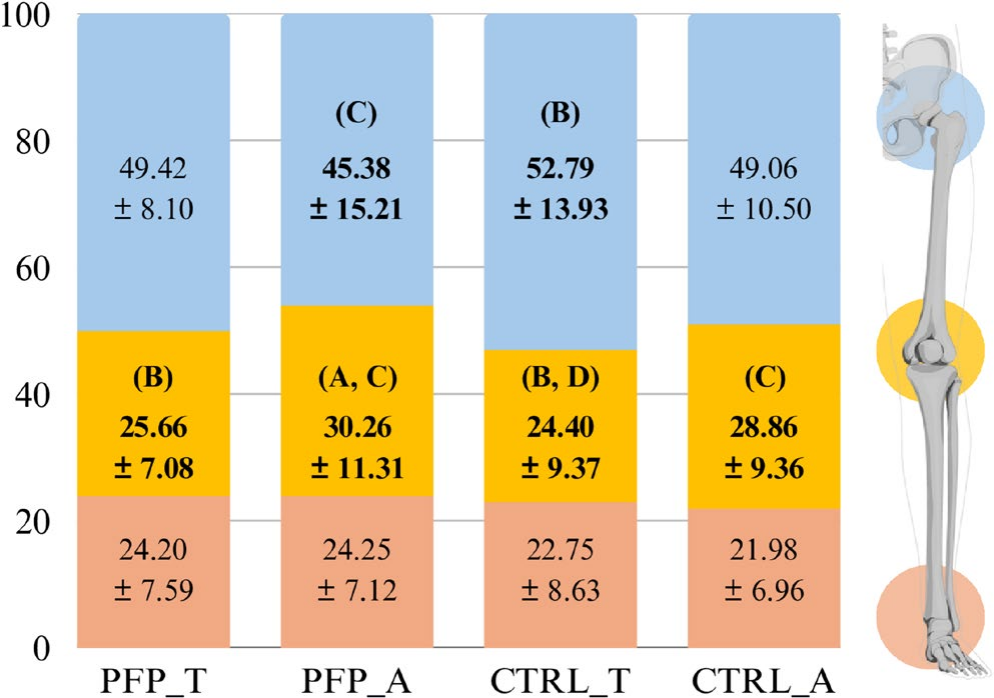
Hip, Knee and ankle contribution ratio (%) to total support moment between groups^α^. ^α^Data are reported in % as mean ± SD. Data were analyzed using generalized linear models adjusted for sex. (A) Represents significant difference as compared with PFP_T. (B) Represents significant difference as compared with PFP_A. (C) Represents significant difference as compared with CTRL_T. (D) Represents significant difference as compared with CTRL_A.

PFP_T participants demonstrated a lower knee contribution to the total support moment (*p* = 0.034; 95% CI [−8.85, −0.35]; *d* = −0.49) compared with PFP_A participants (Figure 2), with no significant differences in total support moment, hip and ankle contributions, or patellofemoral stress (*p* > 0.05; Table 3). Compared with CTRL_T participants, PFP_T participants demonstrated a lower total support moment (*p* = 0.026; 95% CI [−1.90, −0.12]; *d* = −0.51) and lower patellofemoral stress (*p* = 0.044; 95% CI [−8.07, −0.11]; *d* = −0.46), but no significant differences when compared with CTRL_A participants (*p* > 0.05). PFP_A participants demonstrated a lower hip (*p* = 0.031; 95% CI [−14.12, −0.70]; *d* = −0.51) and greater knee (*p* = 0.017; 95% CI [1.09, 10.63]; *d* = 0.56) contribution to the total support moment, and lower patellofemoral stress (*p* = 0.037; 95% CI [−10.51, −0.33]; *d* = −0.49) compared with CTRL_T participants, but no significant differences compared with CTRL_A participants (*p* > 0.05). No significant differences were identified for total support moment or ankle contribution to the total support moment (*p* > 0.05). Compared with CTRL_A participants, CTRL_T participants demonstrated a greater total support moment (*p* = 0.028; 95% CI [0.10, 1.74]; *d* = 0.51), lower knee contribution to the total support moment (*p* = 0.039; 95% CI [−8.69, −0.23]; *d* = −0.48), and greater patellofemoral stress (*p* = 0.015; 95% CI [1.00, 9.06]; *d* = 0.56). No significant differences were identified for hip and ankle contributions to the total support moment (*p* > 0.05).

### 3.5 Muscle Strength

PFP_T participants demonstrated no significant differences in maximal isometric torque of the knee extensors, flexors, and hip abductors when compared with PFP_A participants (*p* > 0.05; Table 4). Compared with CTRL_T participants, PFP_T participants demonstrated lower maximal isometric torque of the knee extensors (*p* = 0.010; 95% CI [−59.52, −8.54]; *d* = −0.60), flexors (*p* = 0.046; 95% CI [−26.41, −0.27]; *d* = −0.46), and hip abductors (*p* = 0.017; 95% CI [−29.19, −2.93]; *d* = −0.55), but no differences when compared with CTRL_A (*p* > 0.05). Compared with CTRL_T participants, PFP_A participants presented with lower maximal isometric torque of the knee extensors (*p* = 0.048; 95% CI [−46.69, −0.21]; *d* = −0.46), but no differences when compared with CTRL_A participants (*p* > 0.05). Compared with controls, PFP_A participants demonstrated no significant differences for maximal isometric torque of the knee flexors and hip abductors (*p* > 0.05). Compared with CTRL_A participants, CTRL_T participants demonstrated no differences in maximal isometric torque of the knee flexors and hip abductors (*p* > 0.05).

**TABLE 4:** Knee and hip muscle torque outcomes.

Variables	Groups			
	PFP_T (*n* = 40)	PFP_A (*n* = 37)	CTRL_T (*n* = 38)	CTRL_A (*n* = 40)
Maximal isometric torque of the knee extensors, Nm·kg^−1^ × 100	254.67 ± 65.90^c^	265.25 ± 55.96^c^	288.70 ± 44.51^a^, ^b^	270.54 ± 56.86
Maximal isometric torque of the knee flexors, Nm·kg^−1^ × 100	141.02 ± 34.47^c^	145.77 ± 28.47	154.36 ± 21.70^a^	144.11 ± 25.61
Maximal isometric torque of the hip abductors, Nm·kg^−1^ × 100	100.58 ± 30.55^c^	103.50 ± 32.00	116.64 ± 27.49^a^	106.96 ± 35.48

*Note:* Data are reported as mean ± SD. Data were analyzed using generalized linear models adjusted for sex.

Abbreviation: Nm·kg^−1^, Newton‐meters per kilogram.

^a^ Represents significant difference as compared with PFP_T.

^b^ Represents significant difference as compared with PFP_A.

^c^ Represents significant difference as compared with CTRL_A.

## 4 Discussion

We aimed to investigate whether people with PFP following knee trauma (e.g., injury or surgery) present with different clinical (patient‐reported outcomes and hop performance), trunk and lower limb biomechanics, and muscle strength features when compared with people with gradual‐onset PFP or pain‐free controls. People with PFP_T demonstrated greater clinical severity (i.e., higher levels of pain and kinesiophobia, and lower self‐reported function) and a more hip‐dominant pattern of movement (i.e., greater trunk and hip flexion) during landing tasks when compared with people with PFP_A. There were also similar impairments in both PFP groups (e.g., worse self‐reported function, SLHD performance, and knee extensor strength) relative to controls (i.e., CTRL_T and/or CTRL_A). These findings highlight that traumatic‐onset PFP does not present with the same clinical and biomechanical profile as gradual‐onset PFP. A history of knee trauma should therefore be considered when assessing people with PFP.

Our findings indicate that PFP symptoms that developed following knee trauma may be associated with greater clinical severity (i.e., worse self‐reported pain and function). Blunt trauma or previous knee surgery have previously been linked to elevated levels of anterior knee pain [13, 31], with post‐traumatic swelling [32], diminished knee confidence [13, 14], and concomitant injuries to surrounding joint structures possible mechanisms [31]. People with PFP_T also reported higher levels of kinesiophobia compared with those with PFP_A. This aligns with previous research indicating that elevated kinesiophobia is associated with greater pain intensity and poorer self‐reported function in people with PFP [33]. Our findings indicate that people with PFP_T may present a distinct clinical profile, characterized by greater severity, compared with those with PFP_A. Clinicians should be aware that a history of knee trauma may warrant more individualized management strategies, particularly those targeting psychological distress [34], when aiming to promote long‐term recovery. Additionally, worse pain and self‐reported function are well known predictors of poor prognosis after treatment in PFP_A population [4]. Future studies are needed to determine whether people with PFP_T would experience a worse prognosis compared with those with PFP_A.

People with PFP_T demonstrated a more hip‐dominant landing pattern compared with PFP_A, characterized by greater trunk and hip flexion angles and reduced knee joint contribution to the lower‐limb support moment. These findings indicate that a history of knee trauma seems to be associated with changes in the movement patterns, potentially reflecting a “quadriceps avoidance” strategy to reduce knee loading [35]. This pattern may be underpinned by higher levels of pain and/or kinesiophobia presented by those with PFP_T as compared with PFP_A. The lower hop‐related performance of people with PFP_T as compared with controls may also reflect this avoidance strategy. Arthrogenic muscle inhibition due to the previous knee trauma could also be associated with this movement pattern as it limits voluntary muscle activation [36]. Conversely, people with PFP_A presented with no differences in the landing pattern when compared with the CTRL_A, which aligns with recent findings [21]. People with PFP_A presented differences in the landing pattern (e.g., greater contribution of the knee to the TSM) only when compared with trauma‐related groups (i.e., PFP_T and CTRL_T). This supports the interpretation that PFP alone may not directly influence joint contribution during landing tasks [21]. Instead, a history of knee trauma appears to play a key role. Collectively, our findings suggest that people with PFP may present with different biomechanical adaptations depending on the history of knee trauma, which could influence research findings. Our findings raise the hypothesis that people with PFP_T might benefit more from knee‐focused interventions (e.g., knee strengthening) to minimize consequences of the hip‐dominant movement pattern (e.g., quadriceps avoidance). However, this needs to be tested in future clinical trials.

Some comparisons were only statistically different to one pain‐free group. Both PFP groups presented with lower knee extensor torque as compared with CTRL_T, but not when compared with CTRL_A. The CTRL_T group also presented with a more hip‐dominant landing strategy compared with PFP_A. Differently from the PFP_T group, it is likely that this movement pattern happened due to the greater hop‐related performance presented by the CTRL_T as compared with the other three groups. This is supported by the findings of greater overall lower‐limb strategy and patellofemoral joint stress presented by the CTRL_T group (i.e., greater lower limb and PFJ loading, respectively). As knee strength has been previously associated with greater hop‐related performance [37], the greater knee extensor torque presented by the CTRL_T group as compared with the PFP groups could be associated with these findings. Greater hip extensor and ankle plantar flexor strength could also be associated with the greater SLHD performance and hip‐dominant landing [38]. However, they were not measured in our study and future endeavors are warranted. In addition, plantar flexor strength was not assessed, which may also be considered given the contribution of the ankle in landing tasks [39]. These findings indicate that the history of knee trauma seems to be associated with changes in trunk and lower limb biomechanics and muscle strength in pain‐free individuals, which may affect how research and clinical assessments are interpreted.

### 4.1 Strengths and Limitations

This is one of the largest cross‐sectional studies in the PFP field and, to the best of our knowledge, the first to compare traumatic‐onset PFP and gradual‐onset cases. We have included various types of knee trauma and surgical procedures, which were not analyzed separately. This may have introduced heterogeneity to our data. Based on our findings, future studies with more restrictive temporal criteria and stratification are now warranted. The cross‐sectional design means it is neither possible to infer causal relationships between the observed changes and the presence of knee trauma, nor whether previous knee trauma directly contributed to the onset of symptoms. There is an imbalance of males and females across our groups, particularly in the CTRL_T group, but we mitigated this by including sex as a covariate in the analysis of the biomechanics variables (i.e., kinematics, kinetics, and muscle torque). Our biomechanical analysis was limited to a landing task. While this task places high loads on the patellofemoral joint and may exacerbate PFP symptoms [40], it may not represent other painful activities that provoke symptoms, such as squatting and running [1]. Our sample consisted of young adults and, therefore, the findings may not be generalizable to other populations where PFP is also high prevalent (e.g., adolescents).

## 5 Perspective

People with PFP_T appear to present a worse clinical condition, a more hip‐dominant landing strategy, and similar muscle strength deficits compared with those with PFP_A. Some impairments (e.g., landing‐related mechanics and muscle strength) were only evident when compared with CTRL_T, who also had differences compared with CTRL_A (e.g., clinical outcomes and landing pattern strategy). These findings highlight the importance of considering previous knee trauma when assessing people with and without PFP.

## Funding

This work was supported by the São Paulo Research Foundation (FAPESP) (Grant Nos. 2021/09393‐1, 2022/06403‐9, and 2023/11083‐6) and Coordenação de Aperfeiçoamento de Pessoal de Nível Superior ‐ Brasil (CAPES) ‐ Finance Code 001.

## Conflicts of Interest

The authors declare no conflicts of interest.

## Supporting information


**Table S1:** Mean differences, 95% confidence intervals, and *P* values for between‐group comparisons for participants' characteristics.
**Table S2:** Mean differences, 95% confidence intervals, and P values for between‐group comparisons for clinical variables.
**Table S3:** Mean differences, 95% confidence intervals and P values for between‐group comparisons for trunk and lower limb biomechanics outcomes.
**Table S4:** Mean differences, 95% confidence intervals and P values for between‐group comparisons for knee and hip muscle torque outcomes.

## Data Availability

The data that support the findings of this study are available from the corresponding author upon reasonable request.
