# Supplementary material for: Patellofemoral Pain After Knee Trauma: Exploring Clinical, Biomechanical, and Muscle Strength Variables
Source: Scand J Med Sci Sports. 2026 Apr 15;36(4):e70274. doi: 10.1111/sms.70274 (PMC13081765; doi:10.1111/sms.70274)
Supplement: Supplementary file 1 — Table S1: Mean differences, 95% confidence intervals, and P values for between‐group comparisons for participants' characteristics. Table S2: Mean differences, 95% confidence intervals, and P values for between‐group comparisons for clinical variables. Table S3: Mean differences, 95% confidence intervals and P values for between‐group comparisons for trunk and lower limb biomechanics outcomes. Table S4: Mean differences, 95% confidence intervals and P values for between‐group comparisons for knee and hip muscle torque outcomes. [file SMS-36-e70274-s001.docx]

TABLE S1.

Mean differences, 95% confidence intervals, and *P* values for between-group comparisons for participants’ characteristics.

|  | | | | | Mean Difference (95% CI) | | | | | *P* Value | | | | |
| --- | --- | --- | --- | --- | --- | --- | --- | --- | --- | --- | --- | --- | --- | --- |
|  | PFP_T × PFP_A | PFP_T × CTRL_T | PFP_T × CTRL_A | PFP_A × CTRL_T | | PFP_A × CTRL_A | CTRL_T × CTRL_A | PFP_T x PFP_A | PFP_T x CTRL_T | | PFP_T × CTRL_A | PFP_A × CTRL_T | PFP_A × CTRL_A | CTRL_T × CTRL_A |
| Age, y | 0.59 (-1.23 to 2.41) | 0.46 (-1.63 to 2.55) | 1.25 (-0.57 to 3.07) | -0.13 (-2.10 to 1.84) | | 0.66 (-1.02 to 2.34) | 0.79 (-1.18 to 2.76) | 0.520 | 0.662 | | 0.176 | 0.896 | 0.435 | 0.427 |
| Body mass, kg | 7.41 (-0.15 to 14.97) | 3.80 (-2.63 to 10.23) | 6.34 (-1.00 to 13.68) | -3.61 (-9.91 to 2.69) | | -1.07 (-8.39 to 6.25) | 2.54 (-3.61 to 8.69) | 0.054 | 0.243 | | 0.089 | 0.257 | 0.772 | 0.413 |
| Height, cm | 1.77 (-2.12 to 5.66) | -3.68 (-7.52 to 0.16) | 0.76 (-3.09 to 4.61) | **-5.45 (-9.13 to -1.77)** | | -1.01 (-4.72 to 2.70) | **4.44 (0.78 to 8.10)** | 0.367 | 0.060 | | 0.696 | **0.004** | 0.589 | **0.018** |
| BMI, kg/m^2^ | 2.07 (-0.22 to 4.36) | **2.32 (0.47 to 4.17)** | **2.20 (0.21 to 4.19)** | 0.25 (-1.71 to 2.21) | | 0.13 (-1.97 to 2.23) | -0.12 (-1.74 to 1.50) | 0.076 | **0.015** | | **0.031** | 0.800 | 0.902 | 0.883 |

PFP, patellofemoral pain; PFP_T, group with PFP and a history of knee trauma; PFP_A, group with gradual-onset PFP; CTRL_T, pain-free group with a history of knee trauma; CTRL_A, pain-free group without a history of knee trauma; F, females; M, males; BMI, body mass index

TABLE S2.

Mean differences, 95% confidence intervals, and *P* values for between-group comparisons for clinical variables.

|  | Mean difference (95% CI) | | | | | | *P* Value | | | | | | |
| --- | --- | --- | --- | --- | --- | --- | --- | --- | --- | --- | --- | --- | --- |
|  | PFP_T × PFP_A | PFP_T × CTRL_T | PFP_T × CTRL_A | PFP_A × CTRL_T | PFP_A × CTRL_A | CTRL_T × CTRL_A | | PFP_T x PFP_A | PFP_T x CTRL_T | PFP_T × CTRL_A | PFP_A × CTRL_T | PFP_A × CTRL_A | CTRL_T × CTRL_A |
| Worst pain in the last month (VAS) | **9.93 (1.64 to 18.22)** | NA | NA | NA | NA | NA | | **0.020** | NA | NA | NA | NA | NA |
| Duration of PFP symptoms (months) | 1.80 (-18.87 to 22.47) | NA | NA | NA | NA | NA | | 0.863 | NA | NA | NA | NA | NA |
| Time since knee traumas (months) | NA | -3.74 (-22.84 to 15.36) | NA | NA | NA | NA | | NA | 0.698 | NA | NA | NA | NA |
| Kinesiophobia (TSK) | **4.08 (1.29 to 6.87)** | **4.65 (2.06 to 7.24)** | **5.34 (2.64 to 8.04)** | 0.57 (-2.30 to 3.44) | 1.26 (-1.72 to 4.24) | 0.69 (-2.09 to 3.47) | | **0.005** | **0.001** | **< 0.001** | 0.694 | 0.402 | 0.623 |
| Self-reported function (KOOS-PF) | **-9.62 (-17.02 to -2.22)** | **-29.35 (-35.50 to -23.20)** | **-33.67 (-39.18 to -28.16)** | **-19.73 (-25.49 to -13.97)** | **-24.05 (-29.10 to -19.00)** | **-4.32 (-7.39 to -1.25)** | | **0.012** | **< 0.001** | **< 0.001** | **< 0.001** | **< 0.001** | **0.006** |
| Single-leg hop distance ([cm/cm] × 100) | -10.39 (-23.69 to 2.91) | **-26.38 (-37.53 to -15.23)** | **-13.81 (-24.48 to -3.14)** | **-15.99 (-30.53 to -1.45)** | -3.42 (-17.42 to 10.58) | **12.57 (0.61 to 24.53)** | | 0.124 | **< 0.001** | **0.012** | **0.032** | 0.628 | **0.040** |

PFP, patellofemoral pain; PFP_T, group with PFP and a history of knee trauma; PFP_A, group with gradual-onset PFP; CTRL_T, pain-free group with a history of knee trauma; CTRL_A, pain-free group without a history of knee trauma; VAS, visual analogue scale; TSK, Tampa Scale for Kinesiophobia; KOOS-PF, Knee Injury and Osteoarthritis Outcome Score – Patellofemoral subscale

TABLE S3.

Mean differences, 95% confidence intervals and *P* values for between-group comparisons for trunk and lower limb biomechanics outcomes.

|  | Mean difference (95% CI) | | | | | | *P* Value | | | | | |
| --- | --- | --- | --- | --- | --- | --- | --- | --- | --- | --- | --- | --- |
|  | PFP_T × PFP_A | PFP_T × CTRL_T | PFP_T × CTRL_A | PFP_A × CTRL_T | PFP_A × CTRL_A | CTRL_T × CTRL_A | PFP_T x PFP_A | PFP_T x CTRL_T | PFP_T × CTRL_A | PFP_A × CTRL_T | PFP_A × CTRL_A | CTRL_T × CTRL_A |
| Peak trunk flexion, deg | **5.24 (0.41 to 10.07)** | -0.52 (-4.93 to 3.89) | 3.06 (-1.77 to 7.89) | **-5.76 (-10.28 to -1.24)** | -2.18 (-7.15 to 2.79) | 3.58 (-0.98 to 8.14) | **0.034** | 0.815 | 0.211 | **0.013** | 0.385 | 0.122 |
| Peak hip flexion, deg | **5.74 (0.57 to 10.91)** | -0.62 (-5.60 to 4.36) | 4.40 (-0.02 to 8.82) | **-6.36 (-12.08 to -0.64)** | -1.34 (-6.51 to 3.83) | **5.02 (0.04 to 10.00)** | **0.030** | 0.805 | 0.051 | **0.030** | 0.607 | **0.048** |
| Peak knee flexion, deg | 1.49 (-3.64 to 6.62) | 3.99 (-9.24 to 1.26) | -1.88 (-6.67 to 2.91) | -5.48 (-11.09 to 0.13) | -3.37 (-8.50 to 1.76) | 2.11 (-3.14 to 7.36) | 0.565 | 0.134 | 0.437 | 0.056 | 0.195 | 0.426 |
| Peak ankle dorsiflexion, deg | 0.54 (-2.40 to 3.48) | -0.16 (-3.19 to 2.87) | -1.24 (-4.05 to 1.57) | -0.70 (-3.86 to 2.46) | -1.78 (-4.72 to 1.16) | -1.08 (-4.11 to 1.95) | 0.715 | 0.916 | 0.383 | 0.660 | 0.232 | 0.479 |
| Total support moment, Nm/kg | 0.06 (-1.09 to 1.21) | **-1.01 (-1.90 to -0.12)** | -0.09 (-0.89 to 0.71) | -1.07 (-2.25 to 0.11) | -0.15 (-1.25 to 0.95) | **0.92 (0.10 to 1.74)** | 0.918 | **0.026** | 0.824 | 0.074 | 0.786 | **0.028** |
| Hip contribution ratio, % | 4.04 (-1.43 to 9.51) | -3.37 (-8.48 to 1.74) | 0.36 (-3.81 to 4.53) | **-7.41 (-14.12 to -0.70)** | -3.68 (-9.58 to 2.22) | 3.73 (-1.81 to 9.27) | 0.146 | 0.193 | 0.864 | **0.031** | 0.218 | 0.184 |
| Knee contribution ratio, % | **-4.60 (-8.85 to -0.35)** | 1.26 (-2.47 to 4.99) | -3.20 (-6.89 to 0.49) | **5.86 (1.09 to 10.63)** | 1.40 (-3.30 to 6.10) | **-4.46 (-8.69 to -0.23)** | **0.034** | 0.503 | 0.089 | **0.017** | 0.555 | **0.039** |
| Ankle contribution ratio, % | -0.05 (-3.40 to 3.30) | 1.45 (-2.21 to 5.11) | 2.22 (-1.02 to 5.46) | 1.50 (-2.15 to 5.15) | 2.27 (-0.93 to 5.47) | 0.77 (-2.76 to 4.30) | 0.976 | 0.433 | 0.177 | 0.415 | 0.161 | 0.665 |
| Peak PFJ stress, MPa | 1.33 (-3.00 to 5.66) | **-4.09 (-8.07 to -0.11)** | 0.94 (-2.23 to 4.11) | **-5.42 (-10.51 to -0.33)** | -0.39 (-4.77 to 3.99) | **5.03 (1.00 to 9.06)** | 0.542 | **0.044** | 0.556 | **0.037** | 0.860 | **0.015** |

PFP, patellofemoral pain; PFP_T, group with PFP and a history of knee trauma; PFP_A, group with gradual-onset PFP; CTRL_T, pain-free group with a history of knee trauma; CTRL_A, pain-free group without a history of knee trauma; deg, degrees; MPa, megapascal; Nm·kg^-1^, Newton-meters per kilogram

TABLE S4.

Mean differences, 95% confidence intervals and *P* values for between-group comparisons for knee and hip muscle torque outcomes.

|  | Mean difference (95% CI) | | | | | | *P* Value | | | | | |
| --- | --- | --- | --- | --- | --- | --- | --- | --- | --- | --- | --- | --- |
|  | PFP_T × PFP_A | PFP_T × CTRL_T | PFP_T × CTRL_A | PFP_A × CTRL_T | PFP_A × CTRL_A | CTRL_T × CTRL_A | PFP_T x PFP_A | PFP_T x CTRL_T | PFP_T × CTRL_A | PFP_A × CTRL_T | PFP_A × CTRL_A | CTRL_T × CTRL_A |
| Isometric knee extensor peak torque, Nmkg^-1^*100 | -10.58 (-38.45 to 17.29) | **-34.03 (-59.52 to -8.54)** | -15.87 (-43.27 to 11.53) | **-23.45 (-46.69 to -0.21)** | -5.29 (-30.93 to 20.35) | 18.16 (-4.95 to 41.27) | 0.452 | **0.010** | 0.252 | **0.048** | 0.682 | 0.122 |
| Isometric knee flexor peak torque, Nmkg^-1^*100 | -4.75 (-19.17 to 9.67) | **-13.34 (-26.41 to -0.27)** | -3.09 (-16.61 to 10.43) | -8.59 (-20.22 to 3.04) | 1.66 (-10.62 to 13.94) | 10.25 (-0.48 to 20.98) | 0.514 | **0.046** | 0.650 | 0.145 | 0.788 | 0.061 |
| Isometric hip abductor peak torque, Nmkg^-1^*100 | -2.92 (-17.12 to 11.28) | **-16.06 (-29.19 to -2.93)** | -6.38 (-21.12 to 8.36) | -13.14 (-26.86 to 0.58) | -3.46 (-18.84 to 11.92) | 9.68 (-4.69 to 24.05) | 0.683 | **0.017** | 0.391 | 0.060 | 0.655 | 0.184 |

PFP, patellofemoral pain; PFP_T, group with PFP and a history of knee trauma; PFP_A, group with gradual-onset PFP; CTRL_T, pain-free group with a history of knee trauma; CTRL_A, pain-free group without a history of knee trauma; Nm·kg^-1^, Newton-meters per kilogram.
